# The training needs of Turkish emergency department personnel regarding intimate partner violence

**DOI:** 10.1186/1471-2458-7-350

**Published:** 2007-12-13

**Authors:** H Asli Davas Aksan, Feride Aksu

**Affiliations:** 1 Department of Public Health, Ege University Medical Faculty, Izmir, Turkey

## Abstract

**Background:**

Violence against females is a widespread public health problem in Turkey and the lifetime prevalence of IPV ranges between 34 and 58.7%. Health care workers (HCW) sometimes have the unique opportunity and obligation to identify, treat, and educate females who are abused. The objective of this study was to evaluate the knowledge, attitudes, and experiences of the emergency department (ED) staff regarding intimate partner violence (IPV) at a large university hospital in Turkey.

**Methods:**

A cross-sectional study was conducted in a large university hospital via questionnaire. The study population consisted of all the nurses and physicians who worked in the ED during a two month period (n = 215). The questionnaire response rate was 80.5% (41 nurses and 132 physicians). The main domains of the questionnaire were knowledge regarding the definition of IPV, clinical findings in victims of IPV, legal aspects of IPV, attitudes towards IPV, knowledge about the characteristics of IPV victims and abusers, and professional and personal experiences and training with respect to IPV.

**Results:**

One-half of the study group were females, 76.3% were physicians, and 89.8% had no training on IPV. The majority of the nurses (89.5%) and physicians (71.1%) declared that they were aware of the clinical appearance of IPV. The mean of the knowledge scores on clinical knowledge were 8.84 ± 1.73 (range, 0–10) for acute conditions, and 4.51 ± 3.32 for chronic conditions. The mean of the knowledge score on legal procedures and the legal rights of the victims was 4.33 ± 1.66 (range, 0–7). At least one reason to justify physical violence was accepted by 69.0% of females and 84.7% of males, but more males than females tended to justify violence (chi square = 5.96; p = 0.015). However, both genders accepted that females who experienced physical violence should seek professional medical help.

**Conclusion:**

The study participants' knowledge about IPV was rather low and a training program is thus necessary on this issue. Attention must be given to the legal aspects and clinical manifestations of IPV. The training program should also include a module on gender roles in order to improve the attitudes towards IPV.

## Background

Intimate partner violence (IPV) has a deteriorating influence on society by affecting victims, their children, families, and friends, as well as social and financial relationships. Abused females who have poor physical and mental health suffer more injuries and use more medical resources than non-abused females [1-3]. Females who have experienced physical, sexual, or emotional violence suffer a range of health problems, often in silence. Gender-based violence is widely recognized as an important public health problem, both because of the acute morbidity and mortality associated with assault and its longer-term impact on women's health, including chronic pain, gynecologic problems, sexually-transmitted diseases, depression, post-traumatic stress disorder, and suicide [2-4].

Health care workers (HCW) have the opportunity and obligation to identify, treat, and educate females who are abused. Health care institutions can make significant contributions to addressing violence against females by supporting clinicians and victims [4]. As it has been mentioned by different researchers, emergency services have the highest probability of encountering female victims of IPV [5-7]. The prevalence of current IPV among female emergency department (ED) patients has been estimated to be between 2 and 12% and many patients are at high risk for future violence [8]. Universal IPV screening is recommended in the ED, [1,5,8] but Eliot et al. [9] stated that the screening rates are as low as 10%.

Although the health system has a vital role in dealing with IPV victims, there are many barriers to assisting IPV victims in healthcare institutions. These barriers include the lack of proper training of HCW in caring for victims of IPV and the time constraints in the ED. On the other hand, HCW might share the same cultural norms and prejudices with victims or perpetrators of IPV, which would affect their professional attitudes. Moreover, some physicians might think that IPV is a private family matter and not a health issue. In addition, while the resources allocated to this field are inadequate, some HCW might feel desperate, leading them to professional reluctance [1,10,11].

There are a number of approaches to overcome these barriers. Elliot et al. [9] reported that any training in this field makes physicians more likely to screen possible victims. Campbell [12] also proved that a system-change model of IPV training in the ED was effective in improving staff attitudes and knowledge about battered females and in protocols and staff training, as well as patient information and satisfaction.

### Situation in Turkey

Violence against females is a widespread public health problem in Turkey and the lifetime prevalence of IPV ranges between 34 and 58.7% [13-16]. According to the results of different studies, 9.7–36.4% of females have been beaten by their partners, even during their pregnancies [17-19]. The females in Turkey have relatively equal legal rights with males, but they face inequalities both in public and private areas. Although education is a compulsory legal right for all Turkish citizens, in 2000, 19% of females were illiterate and participation of females in the workforce was 25.9 % in 2001 [20]. In particular, considering social-ethical values and the social honor attached to a female's body in Turkish society, although there is not reliable and precise statistical data, practices like 'virginity control' and 'honor murders' (i.e., the murder of a person who has been perceived as having brought dishonor to their family) are not unusual or unexpected [21].

Unfortunately, neither medical nor nursing curricula comprehensively cover IPV-related issues, such as legal rights of females and the medical consequences of IPV and intervention strategies in Turkey. A few collaborative training projects were carried out by different organizations financed by the Ministry of Health and European Union funds after 2004, but very few HCW participated in these programs. Neither clinical guidelines nor specific recommendations with regard to IPV have been implemented.

According to the Turkish Penal Code, reporting of IPV is mandatory for HCW. Additionally, as stated by the Protection of the Family Law, the offenders are subject to various punitive measures, including imprisonment, but even for life-threatening injuries, the reporting rate is very low. The official reporting process is rather complicated in Turkey. In order to write an official report for IPV victims, a public prosecutor's request through the police is obligatory. This process is easier said than done. Besides the possible reasons mentioned above, the widespread social tolerance for violence in police stations, public prosecutor offices, courts, and health care institutions should also be considered in the causality of the low reporting rate in Turkey [13,17]

There are many surveys which have assessed the knowledge, attitude, and practices regarding IPV in different HCW in developed countries [22-24]. Some of the surveys have focused on the identification and management of abused patients and attitudes towards partner abuse screening [22-24]. In Turkey, no study has been conducted in the ED evaluating HCW knowledge, attitude, and behaviors about IPV.

The objectives of the current study were as follows: 1) to evaluate the approach of the ED staff to the definition of IPV in terms of sexual, physical, emotional, and economic violence; 2) to determine the level of knowledge on legal procedures and clinical findings in victims of IPV; 3) to record the attitudes of the ED staff about IPV victims; and 4) to identify barriers to effective intervention for victims of IPV at Ege University Hospital.

## Methods

### Study Design and Population

This cross-sectional study was conducted in the ED of Ege University Hospital. Ege University Hospital is a large institution with 1800 beds and it is one of the most important health service providers and referral centers in western Anatolia. The target population of the study was the staff of the ED. There are three types of physicians who work in the ED. The first group of physicians (n = 5) is the specialists who work permanently in the ED; the second group (n = 12) is the research assistants of the faculty, who work under a two month clinical rotation program; and the third group (n = 154) works as consultant clinicians who are called when required. All nurses are permanent staff of the ED. The study population consisted of all the nurses (n = 44) and the physicians who worked in the ED between September and October 2002.

The response rate was 80.5 % (41 nurses, 47 female physicians, and 85 male physicians). The non-respondents were visited three times, but did not complete the questionnaire.

### Survey Content and Administration

A questionnaire consisting of 120 questions was developed by the researchers. The main domains of the questionnaire were knowledge about the definition of IPV, clinical findings of IPV victim, legal aspects of IPV, attitudes towards IPV, knowledge about the characteristics of the IPV victims and abusers, professional and personal experiences, and training regarding IPV. The paper-based survey was handed to physicians and nurses by one of the researchers. The questionnaire was self-administered by the respondents and the researchers collected the questionnaires within one day. The content of the questionnaire items are given in Table 1.

**Table 1:** Content of questionnaire items grouped under 7 categories

**1**	**Knowledge on definition of IPV**
	**Content: **This part consist of 42 statements grouped under four main titles (sexual, physical, emotional and economic violence) and respondents were asked to evaluate the degree of violence for each of these items,
	**Question format: **Statements rated on a Likert scale (1 = not violence through, 5 = severe violence)
	**Scoring: **For every group of statements under each title, mean scores calculated. Higher scores for definition of IPV indicated that these statements were considered as more severe violence. Low scores showed that the respondents were to perceive the statements lesslikely as violence. Cronbach's alfa = 0,960
**2**	**Knowledge on clinical findings of IPV**
	**Content: **Following a self evaluation question on their knowledge on clinical findings of IPV, a list of health conditions under 4 main categories: chronic conditions (n = 6), acute conditions (n = 12), psychiatric diseases (n = 8), reproductive health(n = 8) adopted from Heise et al. was prepared
	**Question format: **True, false, don't know questions
	**Scoring: **Each correct answer was scored as one point. The maximum score the respondents would take was the total item number of each category (eg. for acute conditions it was 12). The respondents score for each category was then converted to a ten point scale scoring by multiplying the original score by ten and dividing it by the maximum score of that category (eg. if the respondent achieved 6 points from the acute conditions category it was converted to 5 in the ten point scale.) The mean scores of each category were calculated by this way. Cronbach's alfa = 0,924

**3**	**Knowledge on legal aspects of IPV**
	**Content: **Following a self evaluation question on their knowledge on legal aspects of IPV seven statements about legal responsibilities and important headings on reporting procedure was prepared.
	**Question format**: True, false, don't know questions
	**Scoring: **Each correct answer was scored as one point. Mean score was calculated. Cronbach's alfa = 0,703

**4**	**Attitudes towards IPV**
	**Content: **14 statements were prepared about justifications of physical violence that the respondents found acceptable.
	**Question format: **Statements rated on a Likert scale (1 = disagree, 3 = agree)
	**Scoring: **"partially agree" answers were categorized as "agree" in analyses. Cronbach's alfa = 0,905

**5**	**Knowledge about IPV victims and abusers**
	**Content: **Evaluations on seven statements about the general characteristics of victims and abusers were asked.
	**Question format: **Statements rated on a Likert scale (1 = disagree, 3 = agree)
	**Scoring: **Data given as percentages Cronbach's alfa = 0,653

**6**	**Professional and personal experiences**
	**Content: **Frequency of dealing with IPV patients, screening frequency (n = 4), personal experience on having IPV cases in their families were asked.
	**Question format: **Yes/no questions and frequency of screening rated on a Likert scale (1 = every time, 5 = never)
	**Scoring: **Data given as percentages

**7**	**Training on IPV**
	**Content: **Questions on educational background in terms of graduate and in service training on IPV were asked. (n = 5)
	**Question format: **Yes/no questions and multiple choice questions.
	**Scoring: **Data given as percentages

The general content and specific items of the questionnaire were initially derived after a literature review [4,16,25]. After the questionnaire was prepared, a psychiatry professor working with victims of violence evaluated the instrument in terms of the approaches to the definition of violence and attitude sections. At the same time, two forensic medicine specialists and a lawyer who works in this field evaluated the section on the knowledge on legal aspects of IPV and statements written for true/false questions had been prepared based on the emerging issues. The questionnaire was pilot-tested on 10 physicians who work in the Department of Public Health.

There was one open-ended question regarding the barriers to appropriate interventions for IPV victims. The answers were analyzed and categorized using qualitative content analysis. Both of the authors read the statements thoroughly in order to reach a global understanding of the content. Then the authors organized the statements into codes and further into main themes encompassing the initial codes. To ensure reliability, this thematic analysis was done through an iterative consensus-building process in which writing was coded independently. Disagreements about coding were resolved in face-to-face meetings. Tabulations were used to determine frequencies and distribution of differing themes and codes.

Chi square and t-tests were used for statistical analysis. Statistical significance was taken at the 5% level (p < 0.05).

## Results

### Socio-demographic Characteristics

Of the study group, 50.9% were females and 34.1% were married. Of the respondents, 23.7% were nurses and 76.3% were physicians. The mean age of the study subjects was 27.45 ± 4.18 years (range, 21–50 years). The median of the total duration of employment of the respondents was 3 years. The duration of work was one year or less for 19.1% of the respondents and 41.4% of the group had been employed more than three years.

### Knowledge on Definition of IPV

The participants scored 42 statements using a 5-point Likert scale (1 = not violence through, 5 = severe violence). The average of the degree of violence scored by the respondents for all statements was 3.93 ± 0.72 (range, 2.1–5.00). The highest scores were attributed to "forcing to prostitution" and "beating with a thick stick;" "restrictions on dressing," "isolation from friends and family," and "financial restrictions" received the lowest scores.

The effect of gender for each group of scores on each types of violence which was sub-categorized as sexual, physical, emotional, and economic was evaluated. Both genders gave the highest scores to statements about sexual violence and the lowest scores to violence associated with finances. In four categories of violence, females gave higher scores to statements about the severity of violence and there was a significant difference according to gender (Table 2).

**Table 2:** Relation between gender and means of knowledge scores of IPV definition categories

**Categories**	**Gender**	**Occupation**	**N**	**Mean Score**	**SD**	p*
Sexual	Female	Nurse	41	4.76	0.42	**0.001**
		Physician	47	4.82	0.32	
	Male	Physician	85	4.39	0.81	

Physical	Female	Nurse	41	4.18	0.57	**0.02**
		Physician	47	4.35	0.48	
	Male	Physician	85	4.02	0.81	

Emotional	Female	Nurse	41	3.09	1.19	**0.001**
		Physician	47	3.42	1.02	
	Male	Physician	85	2.71	1.17	

Economic	Female	Nurse	41	3.06	1.31	**0.005**
		Physician	47	3.60	1.02	
	Male	Physician	85	2.81	1.31	

(1 = not violence through, 5 = severe violence)

*T test analysis done through males and females (nurses+physicians)

Female physicians, followed by nurses, gave the highest scores in each category.

### Knowledge on Clinical Findings and Legal Procedures

According to the self-declarations of the HCW, 89.5% of the nurses and 71.1% of the physicians declared that they were aware of the clinical appearance of the females who experienced domestic violence. Although the study participants declared that they were aware of the clinical appearance of IPV victims, the mean knowledge scores based on their answers to the questionnaire differed. The mean knowledge scores were 8.84 ± 1.73 (range, 0–10) for acute conditions, 7.85 ± 2.48 (range, 0–10) for psychiatric diseases, 5.01 ± 3.34 (range, 0–10) for reproductive health problems, and 4.51 ± 3.32 (range, 0–10) for chronic conditions. There was no relationship between the mean knowledge scores of the clinical appearance of the IPV victims and the study participants' occupation, gender, marital status, and working years.

The percentage of the participants who declared that they didn't know the legal procedure which should be followed in case of an IPV patient was 78.8%. The mean of the knowledge score on legal procedures and the legal rights of the victims was 4.33 ± 1.66 (range, 0–7). There were frequent mistakes made by the participants. Although there is a need for a public prosecutor's request, 77.3% of the group declared that there was no need for this request in order to write a forensic report in case of an IPV victim and 15.7% declared that they didn't know the answer. Of the study group, 91.9% thought that it was not necessary to define the injuries of the IPV victim in detail so as to enable the victim to ask for her legal rights and 20.9% of the study participants declared that if the woman did not apply through a legal procedure, the findings of violence may not necessarily be recorded. There was no significant relationship between the knowledge scores regarding the legal aspects of the IPV victims and the gender, occupation, age groups, marital status, and years of employment of the study subjects.

### Attitudes towards IPV and Knowledge about IPV Victims and Abusers

The study participants believed the following: 1) females who experienced domestic violence frequently came from the lower socio-economic classes (52%) and had lower education levels (52.4%), 2) males who beat their wives were usually aggressive in all their social relationships (75.6%), and 3) pregnancy would prevent women from being subjected to violence (45.9%).

Of the study participants, 69.0% of females and 84.7% of males accepted at least one reason to justify wife-beating (chi square = 5.96; p = 0.015). On the other hand, despite gender differences, even females thought that wife-beating would be justified in cases when the woman deceives her husband (31.0%), lies to her husband (22.1%), reminds her husband of his weaknesses (12.6%), criticizes the manner of males (16.1%), or fails to care for children (11.5%), but more males than females tended to justify the violence and think that females deserve physical punishment. Gender differences in the justification of violence were significant except for the statement pertaining to "refusal of sexual intercourse." When gender and occupations were considered together, there were significant differences between nurses, female physicians, and male physicians in the justification for violence. Female physicians stated the most positive and encouraging attitudes (Table 3). There was no significant relationship between the attitudes and the occupation, years of employment, and marital status.

**Table 3:** Relation between gender, occupation and attitudes on justification of physical violence (%)

**Women deserve physical punishment under these situations**	**Gender**	**Occupation**	**Not agreed (%)**	**Agreed (%)**	**χ^2^**	**P**
Lying to husband	F	Nurse	37.5	62.5	13.06	0.001
		Physician	60.9	39.1		
	M	Physician	28.6	71.4		

Talking too much	F	Nurse	62.5	37.5	11.02	0.004
		Physician	76.6	23.4		
	M	Physician	47.1	52.9		

Deceiving husband	F	Nurse	27.5	72.5	8.17	0.017
		Physician	44.7	55.3		
	M	Physician	21.2	78.8		

Criticizing the manner of men	F	Nurse	47.5	52.5	11.35	0.003
		Physician	61.7	38.3		
	M	Physician	31.8	68.2		

Envying husband	F	Nurse	62.5	37.5	11.28	0.004
		Physician	70.2	29.8		
	M	Physician	41.7	58.3		

Not keeping her promise	F	Nurse	42.5	57.5	4.91	0,08
		Physician	61.7	38.3		
	M	Physician	42.9	57.1		

Reminding her husband's weaknesses	F	Nurse	50	50	5.46	0.039
		Physician	61.7	38.3		
	M	Physician	38.8	61.2		

Refuse of sexual intercourse	F	Nurse	69.2	30.8	3.77	0.152
		Physician	78.7	21.3		
	M	Physician	68.4	31.6		

Failure in care of children	F	Nurse	37.5	62.5	10.05	0.007
		Physician	57.4	42.6		
	M	Physician	29.4	70.6		

Sometimes women learn with physical punishment because of their former learning experiences	F	Nurse	87.5	12.5	8.13	0.017
		Physician	89.1	10.9		
	M	Physician	79.8	20.2		

### Professional and Personal Experience on Domestic Violence

The percentage of the participants who had at least one professional experience with an IPV victim as their patient was 66.1%. When asked about current screening practices, 63.9% of the study group declared that they included questions about IPV when they worked with an injured patient, but when the frequency of screening examined; only one-fourth of the study group stated that they screened each injury case from this point of view. In addition, a striking finding was that 41.9% of the respondents had at least one of their relatives as an IPV victim.

### Training

Of the study group, 89.8% had no training regarding how to approach the IPV victim professionally. Among the group who had training, 70.9% believed that the training was not adequate to satisfactorily help the victims. The results of Table 4 show the attitudes of the HCW in the management of IPV victims.

**Table 4:** Evaluation of the health personnel's attitudes on the management of IPV victims (%)

	Gender		Not agreed (%)	Agreed (%)	**χ^2^**	p
Women who experienced physical violence must take professional medical help	F	Nurse	5.4	94.6	0.19	0.906
		Physician	4.3	95.7		
	M	Physician	6.1	93.9		

Health professionals can't help domestic violence victims, because they will return to the same social environment	F	Nurse	53.8	46.2	1.17	0.557
		Physician	46.7	53.3		
	M	Physician	43.4	56.6		

Domestic violence is a private issue, and patients are ashamed to talk about it	F	Nurse	28.2	71.8	5.43	0.06
		Physician	37.0	63.0		
	M	Physician	49.4	50.6		

Dealing with IPV means interfering with the privacy of the family	F	Nurse	65.0	35.0	4.13	0.127
		Physician	80.9	19.1		
	M	Physician	64.7	35.3		

Both genders accepted that females who experienced physical violence should receive professional medical help. Of the female physicians, 53.3% thought that HCW could not help victims of IPV because the victims ultimately return to the same social environment. The majority of the participants declared that dealing with victims of IPV requires interfering with the privacy of the family and patients who are ashamed to talk about it. There was not a gender difference of attitudes towards the management of victims of IPV. There was no significant relationship between professional attitude, occupation, years of employment, and age groups.

Participants were asked about the barriers in dealing with a female IPV victim. Barriers were defined by the participants and classified under four categories: 1) social, 2) institutional, 3) related to health staff, and 4) related to the victims (Table 5).

**Table 5:** Classification of barriers defined by the participants

**Social**	**Institutional**	**Related to health staff**	**Related to the victim**
Lack of legal arrangements (n = 29)	Lack of proper place to interview the victim (n = 12)	Lack of training (n = 17)	Hide and endure abuse despairingly (n = 18)
Lack of social support institutions (n = 15)	Lack of multidisciplinary approach (n = 4)	Lack of knowledge on legal aspect of the issue (n = 13)	Turning back to the same environment (n = 12)
Low socioeconomic status of women (n = 12)	Lack of safety measures for health care workers (n = 4)	Time constraints (n = 8)	Afraid of the repeat of abuse (n = 9)
Insufficiency in the juridical system (n = 8)	Lack of social care workers in ED (n = 4)	Heavy workload of health care workers (n = 6)	Lack of knowledge on legal rights (n = 7)
Operational structure of the security forces (n = 6)	Lack of job descriptions and procedures (n = 2)	Health staff can not help (n = 8)	Shame (n = 3)
Cultural structure (n = 6)	Lack of staff (n = 1)	Health staff share common prejudices (n = 5)	
Feudal & traditional families (n = 5)		Health staff experience the same abuse (n = 3)	
Low education level (n = 4)		Need of increased authorization (n = 2)	
Religion (n = 1)		Shame of asking questions about abuse (n = 1)	

## Discussion

For a very long period of time, the attitudes and beliefs about IPV have been identified as a barrier to effective clinical responses by medical professionals. According to the results of the current study, in spite of their relatively higher level of educational, a strikingly large group of HCW justified IPV in certain circumstances and their attitudes towards physical violence were unexpectedly negative.

Easteal and Easteal [26] reported that a physician's attitude regarding etiology (e.g., attributing IPV to a victim's personality) and professional role resistance (e.g., limiting the focus of care to injuries only) militate against effective intervention. The study group was among the key health staff in dealing with IPV victims. The results of the Turkey Demographic and Health Survey (TDHS) of 2003 about attitudes towards physical violence were better than our study, but nevertheless 39% of females accepted at least one reason as a justification for wife-beating [27]. One important reason for this difference may be due to the difference in the wording of the questionnaires.

Our questionnaire included the statement, "deceiving the husband," as a reason for justifying wife-beating. Unfortunately, 72.5% of the nurses, 55.3% of the female physicians, and 78.8% of male physicians declared that they agreed with the justification of violence in the case of this statement. Deceiving the husband is a taboo in Turkey and it is among the most important reasons of honor murders [28]. In a report about honor murders in Turkey, it was stated that for some females, even divorcing was considered immoral and unacceptable [28]. However, our results regarding the attitudes towards violence were similar with the study of Weiss *et al.*[29] who worked with ED HCW. According to their results, only 50% of the group knew that the victim was not responsible for the abuse before their educational intervention. In another study carried out by the International Planned Parenthood Federation affiliates, who participated in a gender-based violence project, 53% of the participants felt that inappropriate behavior of some females provoked their husband's aggression [30].

In this study, female physicians stated the most positive attitudes. Rose and Saunders [31] suggested that female providers may have more empathic attitudes towards victims of IPV. Even when the victims were identified, a physician's attitude about the etiology of battering and their perception of the limited role they should play further mitigated against effective intervention [30,32,33] There is increasing research showing that preventive care services for females rendered by female professionals increases the acceptability and efficiency of medical services [32,34]. The increase in the employment of female HCW could help solve this problem in Turkey.

The efficiency of training programs in managing victims of IPV has been shown in different studies [35-37]. According to the results of previous studies, the content, frequency, and timing of training are as important as the presence of training. For example Elliot *et al.*[9] declared that 41% of the specifically trained physicians who work in the ED stated that they usually forgot to routinely ask about domestic violence. They found "any history of training" made physicians more likely to screen, but training within the previous year had a stronger influence. In their study, Sitterding *et al.*[38] found that receiving lectures during residency training was found to be a significant predictor of screening every patient for spouse/partner violence among respondents. It has been demonstrated that clinicians with specific training in abuse assessment are more likely to suspect and screen for it [39,40].

When the content of the training program in Turkey is considered, the relationship between violence and reproductive health problems and chronic diseases should be emphasized. Since lack of knowledge is a prominent feature for both nurses and physicians, an initiative is needed for developing curricula for both graduate and postgraduate training programs. Training of HCW might have a dramatic effect on diminishing the gender effect on the justification of violence, creating positive attitudes towards the issue and realizing effective interventions for IPV victims.

Barriers to the management of IPV victims defined by our respondents were consistent with the literature [41-43]. Although the most common barrier defined by the group was lack of legal arrangements, at the same time, the knowledge score of the participants about the legal aspects of the issue was not satisfactory. The legal context is complicated and not protective enough for the victims. There are no clear procedures to manage the IPV victims in the ED in Turkey. However, informing the victims about their legal rights and starting the legal procedure right after the incident could be a life-saving intervention. The lack of referral social care centers and lack of social care workers in ED were barriers defined by the respondents. At the time of this study, there was only one shelter for females with a capacity of 24 in a city with 2.5 million inhabitants [44]

The work presented here represents an initial effort to provide basic information about the knowledge and attitudes of HCW about victims of IPV. We did not analyze the validity of the questionnaire, but the main domains of the questionnaire were consistent with most of the domains of Sugg *et al.*[22], except the items about batterers, proper referrals, and written guidelines. In this study, we did not assess domains, such as workplace issues and victim autonomy and knowledge regarding causes of violence (e.g., alcohol and drugs), as mentioned in the comprehensive questionnaire developed by Short *et al.*[24]. In the questionnaire developed by Sugg *et al.*[22], all the domains were self-evaluated and reported and focused in the screening capacity of the HCW [22]. However, in this study, we assessed the actual knowledge on clinical manifestations and legal statements concerning IPV. As is the case in the Short *et al.*[24] questionnaire, our questionnaire did not assess actual behaviors; however, it presented us valuable information prior to developing a training program in the ED in Turkey. There were various limitations in this study, such as the low coverage among the physicians (65.0%) due to the difficulties in approaching the consultant physicians, which was the biggest group. Nevertheless, this study was carried out in one of the largest university hospitals of the country and brought up the main obstacles of the HCW dealing with IPV victims.

## Conclusion

Few HCW feel that they have sufficient training in managing IPV victims and many of them share the common prejudices which hinder them from appropriate interventions. There is a growing need for written procedures and guidelines to assist them in case management. At the same time, there should be continuous and relevant training programs on clinical, legal, and cultural aspects of the problem. In addition, a training program should include a component about gender roles in order to diminish the gender effect on the justification of violence and improve the attitudes of HCW towards IPV. We believe that training programs will guide HCW in the management IPV and underline their professional roles.

## Competing interests

The author(s) declare that they have no competing interests.

## Authors' contributions

HADA and FA participated in the design of the study, data analysis/interpretation, statistical analysis, manuscript preparation, manuscript editing, manuscript revision/review and final version approval. All authors read and approved the final manuscript.

## Pre-publication history

The pre-publication history for this paper can be accessed here:


